# Early Exercise Affects Mitochondrial Transcription Factors Expression after Cerebral Ischemia in Rats

**DOI:** 10.3390/ijms13021670

**Published:** 2012-02-06

**Authors:** Qi Zhang, Yi Wu, Hongying Sha, Pengyue Zhang, Jie Jia, Yongshan Hu, Jianhong Zhu

**Affiliations:** 1 Department of Rehabilitation, Huashan Hospital, Fudan University, Shanghai 200040, China; E-Mails: friday0451@163.com (Q.Z.); wishness@sohu.com (P.Z.); shannonjj@126.com (J.J.); 0510160019@smail.tongji.edu.cn (Y.H.); 2 Department of Sports Medicine and Rehabilitation, Medical College of Fudan University, Shanghai 200032, China; 3 The Yonghe Branch of Huashan Hospital, Fudan University, Shanghai 200436, China; 4 State Key Laboratory of Medical Neurobiology, Fudan University, Shanghai 200032, China; E-Mails: shahongying@gmail.com (H.S.); doctorz@126.com (J.Z.); 5 Department of Neurosurgery, Huashan Hospital, Fudan University, Shanghai 200040, China

**Keywords:** exercise, ischemia, PGC-1, NRF-1, COX IV, neuroprotection

## Abstract

Increasing evidence shows that exercise training is neuroprotective after stroke, but the underlying mechanisms are unknown. To clarify this critical issue, the current study investigated the effects of early treadmill exercise on the expression of mitochondrial biogenesis factors. Adult rats were subjected to ischemia induced by middle cerebral artery occlusion followed by reperfusion. Expression of two genes critical for transcriptional regulation of mitochondrial biogenesis, peroxisome proliferator-activated receptor coactivator-1 (PGC-1) and nuclear respiratory factor-1 (NRF-1), were examined by RT-PCR after five days of exercise starting at 24 h after ischemia. Mitochondrial protein cytochrome C oxidase subunit IV (COX IV) was detected by Western blot. Neurological status and cerebral infarct volume were evaluated as indices of brain damage. Treadmill training increased levels of PGC-1 and NRF-1 mRNA, indicating that exercise promotes rehabilitation after ischemia via regulation of mitochondrial biogenesis.

## 1. Introduction

Stroke is a major cause of mortality and dysfunction, as survivors often have physical and/or mental disabilities [1], which cause an enormous burden. Although progress has been made in clarifying the underlying mechanisms and developing therapeutic interventions in recent years, limited treatment options are available for most types of stroke.

The beneficial effect of exercise on stroke rehabilitation has been demonstrated in animal models [2–5]. Exercise has been shown to decrease stroke risk factors, such as body weight, blood pressure, serum cholesterol, and glucose intolerance. Increasing evidence indicates that exercise initiated early after ischemia is associated with smaller brain infarcts, better neurological function, and higher survival rates. While the positive effects of early exercise on functional outcome after stroke are widely recognized, their underlying mechanisms are poorly understood.

Over the past decades, animal studies have supported the role of exercise in accelerating the rate of mitochondrial biogenesis in skeletal muscle [6,7]. However, few studies have investigated this phenomenon in the brain. One recent study demonstrated altered mitochondrial biogenesis (e.g., mitochondrial DNA, PGC-1, and citrate synthase) in mouse brain regions following eight weeks of treadmill training [8]. Similarly, another rodent study demonstrated that exercise stimulates mitochondrial biogenesis by increasing brain levels of PGC-1 protein via the sirtuin 1 pathway [9]. These studies confirmed that exercise can induce mitochondrial biogenesis in the brain, as well as in skeletal muscle.

Conversely, others have hypothesized that mitochondrial biogenesis might occur naturally after brain injury. Two reports provide evidence supporting this theory, demonstrating mitochondrial biogenesis after hypoxic-ischemic (H-I) brain injury, which ameliorated the damage [10,11]. The authors observed significant changes in behavioral scores and cerebral infarct volume, as well as increases in total mitochondria and levels of mitochondrial DNA, heat shock protein-60, mitochondrial respiratory protein COX IV, and the mitochondrial transcription factors PGC-1, NRF-1, and mitochondrial transcription factor A.

While mitochondrial biogenesis is known to be enhanced after exercise or H-I injury, the effects of exercise on mitochondrial biogenesis after injury are unknown. The present study tested the hypothesis that exercise can alleviate ischemic damage by increasing mitochondrial biogenesis. Specifically, we investigated the effect of five days of post-ischemic exercise on neurological status, expression of mitochondrial-specific transcription factors (PGC-1, NRF-1), mitochondrial protein (COXIV) and infarct size.

## 2. Results and Discussion

### 2.1. Neurological Status

**Figure 1:**
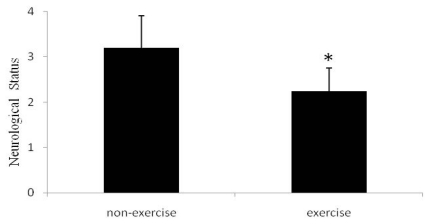
Neurological scores at 5 days after Middle Cerebral Artery Occlusion (MCAO). Sham animals all had scores of zero (data not shown). Relative to non-exercise controls, the rats exposed to exercise had significantly lower neurological scores, indicating improvement, * *p* < 0.05. Data represent mean ± SD. *n* = 6.

A seven-point neurological scale revealed that sham rats had no neurological symptoms. As shown in Figure 1, the rats exposed to ischemia without exercise showed deficits (3.20 ± 0.71), while the rats exposed to ischemia followed by 5 days of post-ischemia exercise demonstrated significantly better neurological statuses (2.14 ± 0.51).

### 2.2. Cerebral Infarct Volume

**Figure 2:**
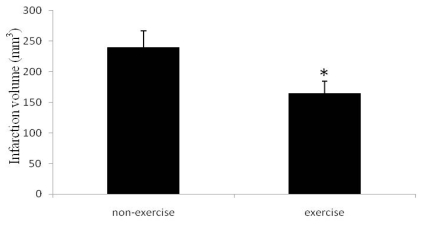
Cerebral infarct volumes. Sham animals all had infarct volumes of zero (data not shown). Relative to non-exercise controls, the rats exposed to exercise had smaller infarct volumes, *p* < 0.05. Data represent mean ± SD. *n* = 6.

None of the sham rats had infarcts. As shown in Figure 2, the rats exposed to ischemia had infarcts (240 mm^3^ ± 26.75), while those exposed to ischemia plus exercise showed significantly reduced infarct volume (165 mm^3^ ± 19.81).

### 2.3. Expression of Mitochondrial-Specific Transcription Factors

**Figure 3:**
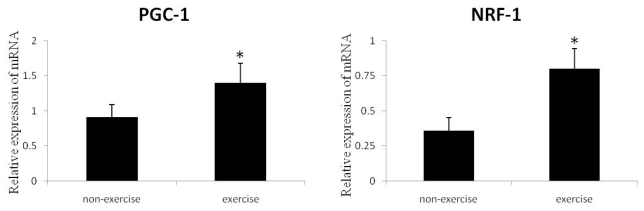
Quantitative RT-PCR analysis of PGC-1 and NRF-1 mRNA in the cortex ipsilateral to MCAO. Relative to non-exercise controls, the rats exposed to exercise had significantly higher levels of mRNA for both mitochondrial biogenesis factors, *p* < 0.05. Data represent mean ± SD and are normalized to GAPDH. *n* = 6.

To determine the effect of exercise on mitochondrial biogenesis after MCAO, we measured the mRNA levels of PGC-1 and NRF-1, which are regulators of mitochondrial biogenesis. As shown in Figure 3, PGC-1 and NRF-1 mRNA expression significantly increased in the exercise group compared to the ischemia without exercise and sham groups.

### 2.4. Expression of Mitochondrial Protein

**Figure 4:**
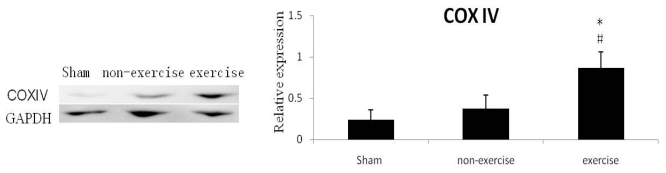
Western blot analysis of COXIV in the cortex ipsilateral to MCAO. Relative to controls, the rats in exercise group had significantly higher levels of COXIV. Optical density values normalized to their respective GAPDH loading control were averaged ± SD and graphed (relative expression). * *p* < 0.05, *versus* sham group respectively, ^#^ *p* < 0.05 *versus* non-exercise group respectively; *n* = 6.

To get more evidence for exercise-induced mitochondrial biogenesis after MCAO, we also detected expression level of mitochondrial protein. COXIV protein is the terminal enzyme on the mitochondrial protein chain, which is only detected in mitochondrial inner membrane fraction. As shown in Figure 4, COXIV protein expression was elevated in the exercise group compared to the other groups.

### 2.5. Discussion

The current study investigated, for the first time, the effects of early treadmill exercise on mitochondrial-specific transcription factors in an ischemic rat model. Five days of treadmill exercise starting at 24 h after MCAO improved neurological function, enhanced mRNA expression of PGC-1, NRF-1 and protein expression of COX IV and reduced lesion volume, relative to non-exercised ischemic controls. These results suggest that early treadmill exercise might promote mitochondrial biogenesis after ischemic injury, thus improving outcome.

Exercise training is well-established to protect against ischemia-induced brain injury [12–15]. In the past, it was thought that patients should engage in post-stroke exercise training only when medically stable. Recent animal and clinical studies have shown that early treadmill training after ischemia can promote functional improvements without exacerbating neuronal tissue loss [16–18]. Previous unpublished work from our lab also showed that very early physical rehabilitation starting 24 h after ischemia yields significant neuroprotection and provides improved neurobehavioral recovery from focal ischemic brain injury in rats. Mechanisms underlying this neuroprotection may include attenuation of pro-inflammatory reactions, brain edema, blood-brain barrier damage, and cognitive and behavioral deficits. In addition, the current study suggests an additional mechanism underlying the neuroprotective effect of early exercise: regulating mitochondrial biogenesis.

In recent years, PGC-1has become one of the most widely-studied proteins in cellular metabolism. It has been recognized as an important regulator of a wide variety of metabolic processes, including mitochondrial biogenesis in skeletal and cardiac muscle, as well as in the brain. PGC-1 initiates the process of mitochondrial biogenesis, and the interaction between PGC-1 and NRF-1 is the first step of this process [7,19–23]. NRF-1 binding sites are located within the promoters of multiple nuclear genes, and interaction of NRF-1 at these sites initiates the synthesis of mitochondrial proteins, including cytochrome *c*, components of the electron transport chain complexes, mitochondrial import proteins, heme biosynthesis proteins, and mitochondrial transcription factor A, which is the final effector necessary for the duplication of mitochondrial DNA [22,24].

In 2011, one study showed significant exercise-induced increases in PGC-1 mRNA expression in the soleus muscle and in a majority of the brain regions, such as the brain stem, cortex, hippocampus, hypothalamus, and midbrain [8]. Another group found that protein levels of sirtuin1 and PGC-1, which were positively correlated with mitochondrial components, were upregulated in the hippocampus by exercise [9]. NRF-1 mRNA and protein expression has also been shown to increase in a rat model of neonatal H-I brain injury [10]. The current data, combined with the observed increased brain mitochondrial DNA, other mitochondrial transcription factors, and mitochondrial protein expression, confirm that mitochondrial biogenesis does indeed occur after brain injury. Furthermore, another report observed neuroprotection associated with upregulation of PGC-1 and NRF-1 in mice subjected to permanent MCAO, further supporting a role for mitochondrial biogenesis after ischemia.

However, no studies have yet examined the effects of exercise training on mitochondrial biogenesis after brain ischemia. The present study observed significant increases in PGC-1 and NRF-1 expression after five days of treadmill exercise initiated 24 h after ischemia. We also detected that levels of the mitochondrial specific protein COX IV increased after exercise. In addition, a reduced infarct volume and improved neurological function were observed in the exercise group. These data provide indirect evidence that mitochondrial biogenesis may be induced by exercise initiated early after brain injury.

The present study has several limitations that should be noted. First, we did not determine the amount of mitochondria and mtDNA. Second, the number of chosen mitochondria biomarker was on the small side. Hence further comprehensive researches are warranted to confirm the conclusions presented herein.

## 3. Experimental Section

### 3.1. Animals and Experimental Groups

Healthy male Sprague-Dawley rats (weighing 250–280 g) were provided by the Shanghai Laboratory Animal Center, Chinese Academy of Sciences, and were housed on a 12:12 hour light/dark cycle with free access to food and water. Animals were randomly assigned to three experimental groups: ischemia with exercise, ischemia without exercise, or sham surgery. The exercise group ran on a rat treadmill (DSPT-202 Type 5-Lane Treadmill; Litai Biotechnology Co., Ltd, China) for 30 min daily for five days after ischemia (post-operation day 1 to 5). On post-operative day 1–2, treadmill velocity was increased over ten-min intervals from 5 m/min to 9 m/min and finally 12 m/min. On post-operative day 3–5, treadmill velocity was 12 m/min for the full 30 min. The treadmill tilt angle remained at 0° throughout the exercise paradigm. Rats in the ischemia without exercise and sham groups were not exposed to the treadmill, but remained in their home cages.

### 3.2. Middle Cerebral Artery Occlusion (MCAO)

All groups were anesthetized with chloral hydrate (350 mg/kg, i.p.). Rectal temperature was maintained at 37 °C throughout the procedure using a circulating heating pad. Ischemia was induced by left MCAO, as previously described [25]. Briefly, to occlude the origin of the middle cerebral artery, a 4-0 surgical nylon monofilament with a silicone tip was advanced from the external carotid artery into the lumen of the internal carotid artery. After 60 min of cerebral ischemia, the filament was withdrawn to allow reperfusion. Rats in the sham group underwent the same surgical procedure without MCAO.

### 3.3. Evaluation of Neurological Status

Neurological status was assessed after recovery from anesthesia and again five days later using a 7-point scale, as previously reported [26]. Scores were assigned as follows: 0, no deficit; 1, failure to extend right forepaw fully; 2, decreased grip of the right forelimb when held by tail; 3, spontaneous movement in all directions, but torso turning to the right side when held by tail; 4, circling or walking to the right; 5, walking only when stimulated; 6, no spontaneous activity; and 7, dead. Only rats with scores of 2–4 after surgery were considered a successful model of ischemia and were randomly assigned to the exercise or no exercise groups.

### 3.4. Measurement of Cerebral Infarction Volume

Infarct volume was analyzed by 2,3,5-triphenyl-tetrazolium chloride (TTC) staining [27]. Rats were anesthetized with chloral hydrate (10%), and the brains were dissected, washed in PBS, and sliced into 2 mm sections. Sections were placed into 2% TTC in PBS at 37 °C for 30 min, and then the TTC solution was replaced with 4% paraformaldehyde fixation buffer for 24 h. Sections were photographed using a digital camera (DC240; Kodak, USA), and infarct size was determined from photographs using NIH image analyzer software (NIH, USA).

### 3.5. RNA Isolation and RT-PCR

Animals were sacrificed after the treadmill exercise regimen ended, and total RNA was isolated from samples of ipsilateral cortex using Trizol reagent according to the manufacturer’s instructions. The integrity and purity of the total RNA was examined by electrophoresis, and the absorbance ratio was at 260 and 280 nm (A260/280). The first strand of cDNA was synthesized from 2–3 μg template RNA using a reverse transcription kit purchased from Takara. RT-PCR of cDNA was performed (ABI PRISM 7500 Sequence Detection System, Applied Biosystems) using the forward and reverse primer sequences shown in Table 1. GAPDH served as an endogenous control. Target mRNA was normalized to GAPDH expression using a comparative critical threshold (Ct) method in which the amount of target was determined relative to the control sample after normalizing to the endogenous control. Specifically, the relative fold change of the target gene expression was expressed as 2^−ΔΔCt^, where ΔΔ*Ct* = Δ*Ct*_test animal_ – Δ*Ct*_calibrator animal_. Three animals in the sham group were randomly chosen to serve as the calibrator sample. The Δ*Ct* was defined as *Ct*_target_ – *Ct*_GAPDH_.

### 3.6. Western Blotting

Ipsilateral cortical tissue was harvested 5 days after MCAO. Cortical protein extracts and Western blotting analysis were performed as previously described [28]. Protein (40 μg) was separated by 15% SDS-polyacrylamide gel electrophoresis (SDS-PAGE) and transferred to PVDF membranes. Primary antibodies were anti-COXIV (Cell Signaling Technology) and anti-glyceraldehyde 3-phosphate dehydrogenase (GAPDH) (Cell Signaling Technology). Membranes were then washed and incubated with secondary antibodies for 1 h at room temperature. Quantification of band intensity (optical density) was carried out on scanned Western blot images using ImageJ software from blots of independent experiments.

### 3.7. Statistical Analysis

All values are reported as mean ± SD. Statistical analyses were performed using SPSS 13.0 statistical software. Multiple comparisons between groups were determined using one-way ANOVA followed by Tamhane multiple comparison post hoc tests. Differences were considered statistically significant at a level of *p* < 0.05.

## 4. Conclusions

Early treadmill exercise induced the expression of mitochondrial factors and protein after ischemic injury, which may indicate mitochondrial biogenesis. While exercise-induced mitochondrial biogenesis appears to be neuroprotective, the specific mechanisms are unclear. Elucidating these mechanisms could suggest targets for treating ischemia-induced brain damage.

## Figures and Tables

**Table 1:** Primer sequences used for PCR.

Gene	Forward primer	Reverse primer
**PGC-1**	GTGCAGCCAAGACTCTGTATGG	GTCCAGGTCATTCACATCAAGTTC
**NRF-1**	TTACTCTGCTGTGGCTGATGG	CCTCTGATGCTTGCGTCGTCT
**GAPDH**	GGGTCAGAAGGATTCCTATG	GGTCTCAAACATGATCTGGG
